# Dura Closure Tactics to Prevent CSF Leakage in Microvascular Decompression Surgery

**DOI:** 10.3390/life15040574

**Published:** 2025-04-01

**Authors:** Hyun Seok Lee, Kwan Park

**Affiliations:** Department of Neurosurgery, Konkuk University Medical Center, Seoul 05030, Republic of Korea; 20220205@kuh.ac.kr

**Keywords:** triple-layer closing technique, microvascular decompression, dural closing technique, postoperative cerebrospinal fluid leakage

## Abstract

(1) Background: Achieving a complete and secure dural closure to prevent cerebrospinal fluid (CSF) leakage is a critical concern in microvascular decompression (MVD). Proper dural closure minimizes complications, such as infections caused by CSF leakage. This study introduces a novel three-step dural suturing method, termed the “triple-layer closing technique”. (2) Methods: From September 2020 to March 2023, a total of 475 patients underwent MVD surgery at our institution, all of whom received dural closure using the triple-layer closing technique. This technique incorporates three layers: Duragen^®^ (synthetic dura, Integra Lifesciences), TachoSil^®^ (collagen matrix, Nycomed), and polymethyl methacrylate (PMMC) bone cement. Postoperative complications, including CSF leakage and infections, were retrospectively analyzed. (3) Results: CSF leakage was observed in five patients (1.1%), all of whom presented with CSF rhinorrhea and radiological evidence of effusion within the mastoid air cells. These patients were successfully treated with lumbar drainage, and none required reoperation. No other postoperative infections or complications were reported. (4) Conclusions: The triple-layer closing technique, utilizing Duragen^®^, TachoSil^®^, and PMMC bone cement, is an effective and reliable method for dural closure. This technique significantly reduces the risk of CSF leakage and surgical site infections, enhancing postoperative outcomes in MVD procedures.

## 1. Introduction

Cerebrospinal fluid (CSF) leakage is a common complication following posterior fossa surgery and can lead to serious conditions, such as headache, meningitis, and cerebral abscess. These complications not only prolong hospitalization but also increase medical costs for patients. In severe cases, CSF leakage can even become life-threatening [1,2,3,4,5,6,7,8,9].

The reported incidence of CSF leakage after neurosurgical procedures ranges from 5% to 30%, with the risk being approximately six times higher in infratentorial approaches compared to supratentorial approaches due to the effects of gravity and patient positioning [10,11].

Achieving watertight dural closure is essential for preventing postoperative CSF leakage [1,12,13]. To address this issue, numerous techniques have been developed, and many neurosurgeons, including our team, have explored various methods to reduce the risk of CSF leakage [1,2,3,4,5,9,12,14,15,16,17]. Previously, we introduced the “muscle plugging method,” which proved to be effective [6]. However, due to partial muscle layer resection and advancements in artificial dura materials, we have since developed a new technique. Although placing artificial materials on the inner layer of the dura (brain side) may help prevent CSF leakage, it also increases the risk of postoperative infection. To overcome these challenges, we have introduced a new dural closure protocol—the “triple-layer closing technique”. This method, which does not require muscle resection, incorporates Duragen^®^, TachoSil^®^, fibrin glue, and polymethyl methacrylate (PMMC) bone cement to effectively minimize CSF leakage. In this study, we present the outcomes of this novel technique.

## 2. Materials and Methods

A retrospective analysis of all patients who underwent microvascular decompression (MVD) surgery between September 2020 and March 2023 was conducted. All surgeries were performed with a retromastoid suboccipital craniotomy (RMSOC) by a single surgeon (Kwan Park) at KonKuk University Medical Center in the same manner as the triple-layer closing technique. A total of 475 MVDs were performed, including 427 cases of hemifacial spasm, 47 cases of trigeminal neuralgia, and 1 case of glossopharyngeal neuralgia. Additionally, the study group consisted of 131 males and 344 females, with a median age of 58 years (range, 19–82 years) (Table 1).

**Figure 1:**
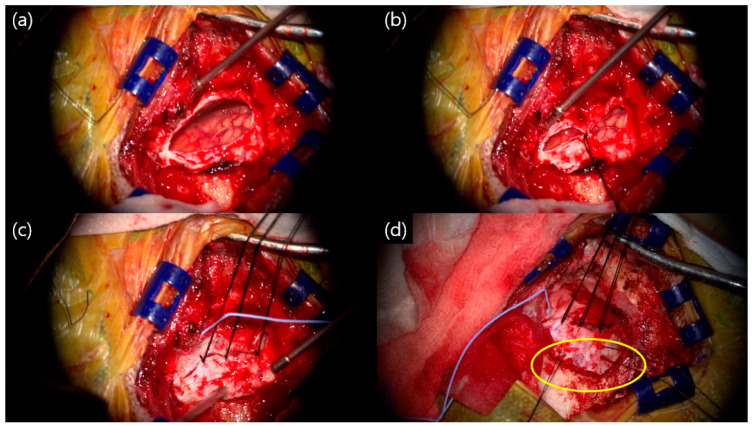
Intraoperative findings, left side hemifacial spasm: (**a**) after neurovascular decompression, before dura closing; (**b**) first suture performed in the middle of the durotomy; (**c**) suture of the remaining unsutured site; (**d**) dura tagging suture (yellow circle) to prevent epidural hematoma.

The triple-layer closing technique (TLCT) is a suture method that prevents the leakage of CSF in three layers on the outside of the dura mater. In this procedure, first, a suture was performed in the middle of the durotomy site (Figure 1a,b). Next, the middle part of the remaining unsutured part was sutured (Figure 1c). If the dural incision was large or a counter incision was made to secure the field of surgical view, 1 or 2 more sites were sutured. To prevent the occurrence of epidural hematoma, a dural tagging suture was performed on the medial side and superior side of the craniotomy site (Figure 1d).

**Figure 2:**
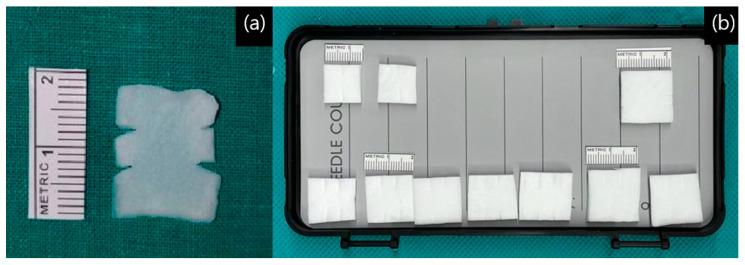
Ready for use, Duragen^®^: (**a**) make two slits, soaked in water; (**b**) cut into different sizes (2 slits, 3 slits, etc.) before soaking in water.

**Figure 3:**
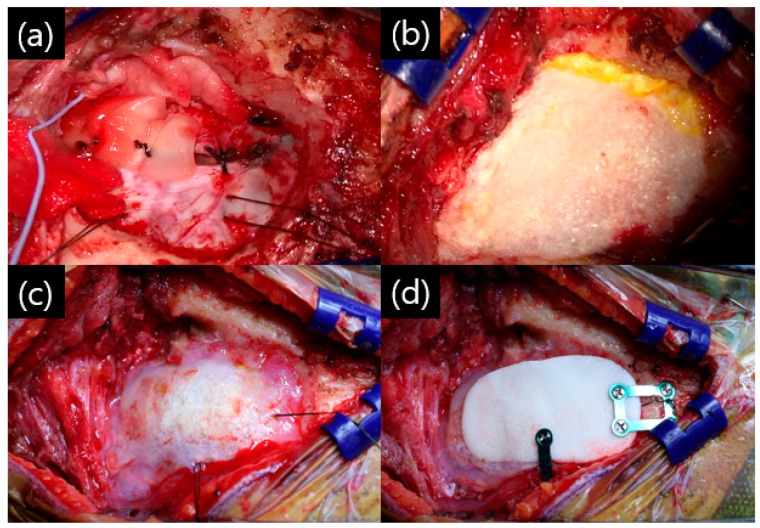
Intraoperative findings of the triple-layer closing technique (TLCT): (**a**) first layer and primary dural suture was performed with Duragen^®^; (**b**) after suturing of the dura, the craniotomy site was covered with TachoSil^®^; (**c**) fibrin glue was sprayed on the TachoSil^®^ layer; (**d**) polymethylmethacrylate (PMMC) bone cement was made to fit the size of the craniotomy site, followed by fixation with a titanium plate and screw.

This suture was later fixed to the bone flap. After tagging the sutures, dural suturing was performed on the remaining unsutured site, usually starting from the caudal side. Prior to insertion, Duragen^®^ was trimmed to the appropriate size. To facilitate tying, slits were made on either side of the graft rather than using a rectangular piece. Typically, two slits were made, but depending on the size of the suture site, one or three slits could be used (Figure 2a,b). After preparation, Duragen® was inserted between two sutures placed at each site before tying (Figure 3a). 

Depending on the size of the suture site, one or more sutures may have been performed. Sutures were performed from the caudal side to the cranial side in turn, especially before the last suture, to ensure sufficient filling of the saline inside the dura. This was carried out to prevent complications, such as subdural hemorrhage caused by a bridging vein injury and the consequently large outflow of CSF during surgery. After completing the dural suturing, the surgeon checked whether there was any leakage of CSF and placed the fibrin sealant, TachoSil^®^, on top of the layer. The TachoSil^®^ sealants were large enough to cover the entire craniotomy site (Figure 3b). TachoSil^®^ is spongy collagen, which is coated with the human clotting factors thrombin and fibrinogen. It can be easily applied to the desired surgical site, and when in contact with blood or other body fluids or saline, it adheres firmly to the applied surface [18]. This is absorbed by the body within a few weeks. Next, Tisseel fibrin glue (Biotek Pharma, Baxter Healthcare Ltd., Thetford, Norfolk, UK) was applied over that layer (Figure 3c). Finally, polymethylmethacrylate (PMMA) bone cement was made to fit the size of the bone flap and fixed with a titanium screw and plate (Figure 3d).

**Figure 4:**
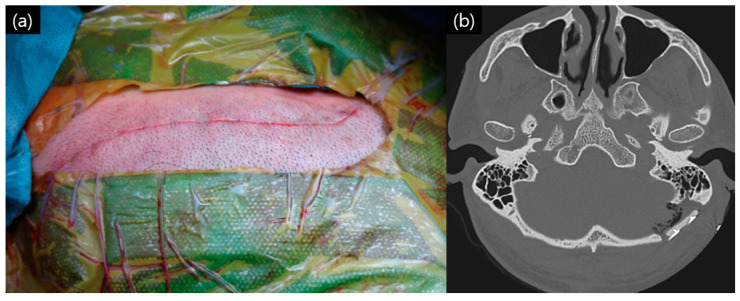
Completed closure: (**a**) operation site after subcutaneous layer suture; (**b**) immediate postoperative temporal bone computed tomography (CT) of the patient.

The previously performed dural tagging suture was tagged to the titanium plate, with the bone cement not layered too thickly. After this process was completed, the muscle layer, subcutaneous layer, and skin layer were sutured to complete the surgery (Figure 4a,b).

The patients were discharged after an average of 5 days of postoperative care. During the hospitalization period, the patients were closely monitored for signs and symptoms of CSF leakage, such as the pseudomeningocele of the surgical site, otorrhea, and rhinorrhea. Among the 475 cases, mastoid air cells were opened in 276 cases (58.1%) during the retrosigmoid approach. When mastoid air cells were encountered intraoperatively, meticulous closure using bone wax was performed both before opening the dura and after completing the dural closure. Preoperative temporal bone CT was used in all patients to evaluate the extent of mastoid pneumatization and to plan for proper sealing strategies if air cells were found to be well-developed.

## 3. Results

Out of a total of 475 patients, who received MVD surgery after the application of the multi-layer closing technique with the “triple-layer closing technique” method for dural sealing, 5 patients (1.1%) had a definite CSF leakage.

The primary clinical presentation was suspicious for CSF leakage in 18 patients (3.8%), there was trigeminal neuralgia in 1 patient and hemifacial spasm in 17 patients. Of those 18 patients, 5 (1.1%) had definite CSF leakage after bed rest and intensive observation (Table 2).

All of these five patients had symptoms of continuous postural rhinorrhea, conductive hearing difficulty. This was a “paradoxical rhinorrhea” in which the CSF from the subarachnoid space exits through the dural defect and passes through the mastoid air cells to the nasal cavity via the Eustachian tube of the middle ear. In these five cases, the presence of opened mastoid air cells may have contributed to the observed CSF leakage, indicating a potential risk factor that warrants further investigation. In all five patients, a lumbar drain catheter was inserted to treat via drain the CSF. The lumbar drainage catheter maintained in the subarachnoid space for an average of 5.2 days (range: 4–6 days). As a result, the average length of hospital stay for these five patients was extended to 14.8 days (range: 13–19 days). All of these patients resolved their symptoms without surgery for permanent CSF diversion, such as a ventricular peritoneal or lumbar peritoneal shunt, or dural repair surgery.

There were no cases of subdural hematoma (SDH) or epidural hematoma (EDH), and there were no mortalities related to surgery. In addition, there were no cases of secondary surgical site infections, such as postoperative wound abscess bacterial meningitis or intracranial abscess (Table 3).

There were no postoperative infections associated with the use of PMMA bone cement or non-absorbable bone wax, and there were no adverse reactions, including allergic reactions, associated with PMMA bone cement and non-absorbable bone wax.

## 4. Discussion

Due to the recent development of artificial dural substitutes, primary watertight dural suturing is not emphasized any more than before, but it is still important in preventing CSF leakage [1,12,13]. Although dural closing methods as well as artificial dural substitutes have been advanced, CSF leakage is still one of the most common complications of posterior fossa surgery, especially MVD surgery [3,6]. Reviewing the existing studies published on MVD, the incidence of CSF leakage after MVD has been reported to be in the range of 0.9–12% of all patients [6,7,19,20,21,22,23]. The cause of this complication is known to be related to the high hydrostatic pressure in the posterior fossa, caused by posture [10,11]. Therefore, it is necessary to withstand high pressure with watertight dural occlusion to prevent CSF leakage after the retrosigmoid suboccipital approach.

We previously published a “plugging muscle” method in 2007, which is a dural suture method applied after MVD surgery [6]. In this method, after the excision of the posterior neck muscle, the muscle piece is inserted at the dural suture site when tying. The “plugging muscle” method that we previously reported showed relatively good clinical results, with CSF leakage occurring in 2 out of 678 (0.3%) patients [6]. The disadvantage of the collagen matrix (Duragen^®^) is the extra cost (USD 100–150 for our suturing technique size) compared to the “plugging muscle” method. Although this may limit its use, it is worth consideration as an alternative surgical option. Lesser occipital nerve damage from muscle resection can cause postoperative headache and subcutaneous dimpling from muscle atrophy [1,24,25]. In addition, another critical concern with the plugging muscle method was the potential risk of infection involving the implanted autologous tissue. Although rare, infection in such cases could lead to the development of a subdural abscess, which is known to have significantly higher morbidity and mortality compared to an epidural abscess. Given the potentially life-threatening nature of subdural abscesses, we aimed to develop a technique that avoids placing organic tissue near the dural defect. The triple-layer closing technique (TLCT) addresses this concern by using synthetic and absorbable materials, thereby minimizing the risk of deep-seated infections, while maintaining an effective multi-layer closure. Therefore, the triple-layer closing technique has been designed to seal the dura tightly and prevent foreign substances from entering the dura, causing infection and inflammation. The use of synthetic materials (Duragen^®^, TachoSil^®^) avoids risks associated with harvesting autologous tissues, while gentamycin-impregnated PMMA bone cement provides local antibiotic coverage. In addition, secure dural sealing significantly reduces infection risks related to CSF leakage, such as meningitis or abscess formation, as confirmed by our infection-free outcomes.

In addition to our previous study, several studies on CSF leakage have continued until recently. Inoue et al. reported the application of a dural suture method after MVD surgery in 120 patients [1]. Their 120 patients composed of 60 patients in the collagen matrix (Duragen^®^) group and 60 patients in the fascia flap group. CSF leakages were observed in 3.3 of the collagen matrix group and 5.0% of the fascial group. The difference between their study and ours is that the symptoms were expressed as pseudomeningocele rather than rhinorrhea. Postoperative infections occurred in four patients, a total of 3.4%, with 1.7% and 5.1% in the collagen matrix group and fascia flap group, respectively. Their use of Duragen^®^ was similar to our technique, but the difference is that it was placed by inlay and outlay, and the suture was not meticulous [1].

Khan et al. also reported a dural closing technique after MVD surgery; they analyzed 134 patients who underwent dural closure using Duraguard^®^ products and Hystacryl glue and found a 3.7% frequency of CSF leakage. One of the other reports involved fleece-bounce sealing of the dura with an artificial material [26]. Tanrikulu et al. reported this method, which involves suturing the inside and outside of the dura with artificial materials in a sandwich-like manner [16].

In particular, there was a review paper of the use of TachoSil^®^, a collagen-bound fibrin sealant that we also used. Carretta et al. reported the use of a collagen-bound fibrin sealant (TachoSil^®^) for dural closure in cranial surgery [27]. A total of 662 patients were included in the study, compared 310 in the using of TachoSil^®^ group with 352 in the non-using of TachoSil^®^ group. The dural closure method was primary sutured in all patients (by PDS^®^, Prolene^®^ or Vicryl^®^, Ethicon Inc., Somerville, NJ, USA) with and without TachoSil^®^ based on the responsible operator’s choices. There was no statistical difference in overall complications (*p*-value = 0.96), including CSF leakage (*p*-value = 0.91), wound infection (*p*-value = 0.91), and meningitis (*p*-value = 0.64), when comparing the TachoSil^®^ group to the non-use group. The conclusion of this systematic review was that the use of this fibrin sealant does not reduce the postoperative complications associated with CSF leakage [27]. However, the difference between our closing method is that they applied the TachoSil^®^ directly over the primary suture site, whereas we proceeded the primary suture with Duragen^®^, and we added another layer of TachoSil^®^, oversized to match the craniotomy size.

PMMA bone cement was first used in the dental department and then in the orthopedic department in the 1940s. PMMA is a type of acrylic plastic, which is formed by combining a powder with a liquid monomer. When this powder is mixed with liquid, an exothermic reaction occurs, which reaches temperatures of 48–56 degrees Celsius in vivo [28].

PMMA does not adhere to bone or metal on its own, so it needs to be fixed with titanium plates and screws; because of the relatively high temperatures generated, the exothermic reaction of this bone cement must be fixed to the skull after the exothermic reaction is complete to prevent tissue damage. Currently, one of the most rapidly increasing uses of PMMA in the orthopedic department is as delivery carrier for antibiotic treatment of infection cases [29]. Commercially usable antibiotic impregnated PMMA contains prophylactic level of antibiotics. For cases in which more antibiotics are needed therapeutically, PMMA bone cement may be mixed with vancomycin or other antibiotics [29], but in our cases, we used PMMA with prophylactic concentrations of gentamycin and did not have any problems with infection. Our surgical approach is to drill all of the bone flap in the surgical site rather than take the bone flap out in one piece with a saw. Since this retromastoid area is a less cosmetically important area for detailed curvature (less visible) and can be more protected from injury of the sigmoid sinus, especially in the delicate handling of the mastoid emissary vein, it may be a good option to use a bone drilling (craniectomy) method and use PMMA bone cement composed with prophylactic antibiotics for closure.

There have been many reports of CSF leakage in MVD and posterior fossa surgery. The dural suture materials used in these reports range from autologous muscle and fascia to artificial dura mater and collagen matrix, and the CSF leakage rate varies from 0.3% to 27% (Table 4). This shows that all the authors, including us, are trying hard to prevent CSF leakage and are also trying a variety of new dural closure methods. Recently, Chibbaro et al. [30,31] introduced a modified C-shaped skin incision and muscle flap approach in retrosigmoid surgery, demonstrating superior outcomes compared to traditional linear or S-shaped incisions. Their studies reported significantly lower postoperative complications, including CSF leakage, wound infections, and postoperative retroauricular pain. Furthermore, anatomical investigations confirmed that this method effectively preserved critical structures, such as the lesser occipital and greater auricular nerves, thereby potentially reducing chronic postoperative pain [31]. Considering studies with more than 1000 cases, in a 1996 paper by Zanetta Barker et al., there was CSF leakage in 20 (1.5%) of 1336 cases of MVD surgeries in 1185 patients with trigeminal neuralgia [32]. In a paper published by our team in 2016 analyzing 2040 cases of MVD, 100 patients presented with middle ear effusion, 12 patients presented with CSF rhinorrhea, and 7 patients presented with both symptoms. Eight of these patients were treat with a CSF drain via lumbar drain catheter [33]. In 2017, Zhao et al. published a paper analyzing the complications of 1548 patients who underwent MVD surgery for hemifacial spasm. In the surgical technique, there is no detailed mention of the dural suture method, but it is mentioned that the bone waxing of the mastoid air cell was thorough. Wound infection occurred in 8 patients (0.52%), and CSF leakage occurred in 24 patients (1.6%). All 24 were treated with CSF drainage via a lumbar drainage catheter. One study that stands out among the others is a study that found that, in 86 patients who underwent the retromastoid suboccipital approach, the primary suturing of the dura without any other material resulted in no CSF leakage. Venable et al. reported no CSF leakage in surgical outcomes for MVD (50 cases), tumors (31 cases), cavernous malformation (2 cases), and intracranial abscess (1 case). The author advocates for caution when handling the dural flap intraoperatively [34]. However, the study has the limitation that the total number of cases is small, less than 100.

In addition, studies that included comprehensive posterior fossa surgery seemed to have a relatively higher rate of CSF leakage compared to studies that analyzed only MVD surgery, which may be the reason why the craniotomy size and durotomy size for posterior fossa tumors should be relatively larger than for MVD. In the case of posterior fossa meningioma, there may be adhesions to the dura mater, and a large defect in the dura mater may occur during the resection of the meningioma. In vestibular schwannomas, intradural internal auditory canal drilling is sometimes performed, which may result in CSF leakage. In rare cases, trauma to the posterior fossa can cause a defect in the dura mater, which is thought to be more likely to result in CSF leakage.

Occasionally, there are reports of natural dural defects found in the posterior fossa dura that are not related to the craniotomy procedure. Yamazki et al. analyzed 593 patients who underwent MVD surgery for trigeminal neuralgia and hemifacial spasm for the presence of a natural dural defect of posterior fossa not created during craniotomy [42]. They reviewed surgical records, checked microscopic surgical videos to confirm, and reported that 6 of 593 patients (1.01%) had natural dural defects. They reported that only left-sided lesions were significantly associated with dural defect in univariate (*p*-value = 0.0165) and multivariate (*p*-value = 0.0108) statistical analyses [42]. In our dural closing technique, we have a second layer that covers the collagen matrix, the TachoSil^®^, and as mentioned in methods, we cover it first in multiple pieces and then finally in one large piece to fit the craniotomy size [Figure 2b]. Because our “TLCT” seals all exposed dura, it seems to be effective in preventing “natural dural defects”.

In our study, mastoid air cells were opened in 276 (58.1%) of the 475 cases. All of our surgical approaches perform bone work in the lateral direction until the sigmoid sinus is visible, the difference between this air cells opening is due to differences in the shape of each patient’s skull. The opening of air-filled parts inside the skull, such as the frontal sinus of the frontal bone or the mastoid air cells of the temporal bone, are notable risk factors for CSF leakage. To prevent this risk factor as much as possible, we performed bone waxing once before opening the dura and again after suturing the dura. We also performed a temporal bone computed tomography (CT) scan in all patients before surgery to determine the development of mastoid air cells. Of course, the advantage of temporal bone CT is that it can also check the thickness of the posterior fossa skull and the location and size of the emissary vein. Furthermore, our findings suggest that the presence of opened mastoid air cells may play a meaningful role in the development of postoperative CSF leakage. In all five cases with confirmed CSF rhinorrhea, mastoid air cells were opened intraoperatively. Although bone wax was applied to seal the air cells in each case, leakage still occurred, highlighting the limitations of sealing alone. These results underline the importance of not only securing a watertight dural closure but also ensuring the thorough identification and closure of mastoid air cells as a crucial step in reducing CSF-related complications. Although the use of a small-pressed muscle flap could be considered as an option for sealing mastoid air cells, we opted to use bone wax alone in all cases to avoid placing organic tissue near the dural defect and to minimize infection risk.

As we mentioned previously, cranioplasty using bone cement itself cannot significantly reduce CSF leakage. Nevertheless, by pressing the bone defect site well, Duragen^®^ on the bottom adheres well to the dura. It also serves to ensure that the adhesive fibrin sealant (TachoSil^®^) adheres well to fine defects of the dura and pinholes caused by needle piercing during suturing.

In our surgical approach, additional drilling on the lateral and inferior margins is often required to expose the sigmoid sinus. This makes the precise replacement of the original bone flap difficult. Therefore, we prefer to use PMMA bone cement for cranioplasty, as it allows a better fit to the craniectomy site and provides effective compression over the dural closure.

The limitation of this study is that the method needs to be continued for a longer period of time to accumulate the number of cases. Due to the small number of patients with cerebrospinal fluid leakage for statistical validation, we recommend that a longer follow-up period and accumulation of case numbers are needed to draw safer conclusions about the rates of cerebrospinal fluid leakage and infection in this TLCT. We suspect that longer follow-up of infections will be needed, especially because of the large number of external materials inserted. In addition, the materials used in the triple-layer closing technique can be relatively costly, which may limit its use in settings with restricted surgical resources. In such cases, alternative methods like our previously reported “muscle plugging method” could serve as a practical and cost-effective option. Comparative studies on clinical outcomes and cost-efficiency between these approaches may be helpful for tailoring strategies to different clinical environments.

## 5. Conclusions

As the number of surgeries increases, complications are inevitably likely to occur, so all surgeons are constantly trying to reduce them and also trying new methods. It has been continually reported that CSF leakage is associated with many fatal complications, and the incidence of CSF leakage is particularly high in posterior fossa surgery.

The “plugging muscle” method previously published by our team in 2007 was an adequate method to prevent CSF leakage. However, we believe that the “triple-layer closing technique” method, which compensates for the problem of resection of the muscle layer and insertion of a foreign body inside the dura, may be a good dural suture method for posterior fossa surgery via retromastoid suboccipital craniotomy. We have performed over 5100 MVD surgeries as of March 2023, still ongoing, and we are now using this method to close the dura. This triple-layer closing technique appears to be effective in reducing the incidence of CSF leakage and other complications.

## Figures and Tables

**Table 1:** Clinical characteristics of patients.

Patients Characteristics	
Median age at MVD (range)	58 (19–82)
Sex (M:F)	131:344
Operation side (left:right)	223:252
Disease (TN/HFS/GPN)	47/427/1
Average hospital days (range)	7.2 (4–18)
Re-do operation	4 (0.8%)
**Outcome associate with complication**	
Suspected CSF leakage	18 (3.8%)
CSF rhinorrhea	5 (1.1%)
CSF diversion via LD catheter insertion	5 (1.1%)
Revision operation	0 (0.0%)
Infection associate with operation	0 (0.0%)
Epidural hematoma	0 (0.0%)

MVD, microvascular decompression; M, male; F, female; TN, trigeminal neuralgia; HFS, hemifacial spasm; GPN, glossopharyngeal neuralgia; CSF, cerebrospinal fluid; LD, lumbar drainage.

**Table 2:** Patients with obvious cerebrospinal fluid leakage.

Sex/Age	Disease	Side	Symptom Duration (Months)	Offending Vessel	Past Medical History
F/39	HFS	Right	18	AICA	Prolactinoma
M/63	HFS	Left	36	AICA-PICA-VA	HTN, dyslipidemia
F/30	HFS	Left	42	AICA-PICA	-
M/38	HFS	Right	24	AICA	Crohn’s disease
F/59	HFS	Right	26	AICA-VA	Dyslipidemia, thyroid cancer

F, female; M, male; HFS, hemifacial spasm; AICA, anterior inferior cerebellar artery; PICA; posterior inferior cerebellar artery; VA, vertebral artery; HTN, hypertension.

**Table 3:** Disease specific characteristics.

Trigeminal Neuralgia	
Total patients	47
Median age at MVD surgery (range)	63.5 (28–80)
Operation side (left:right)	17:30
Sex (male:female)	16:31
Postoperative hearing difficulty	1 (2.1%)
Suspected CSF leakage	1 (2.1%)
Symptom free at 1 month after operation	41 (87.2%)
**Hemifacial spasm**	
Total patients	427
Median age at MVD surgery (range)	58 (19–82)
Operation side (left:right)	227:200
Sex (male:female)	115:312
Postoperative hearing difficulty	32 (7.5%)
Suspected CSF leakage	17 (4.0%)
Symptom free at 1 month after operation	388 (90.9%)
**Glossopharyngeal neuralgia**	
Left side lesion, 65 years old female, with no complication, postoperative pain free	

MVD, microvascular decompression; CSF, cerebrospinal fluid.

**Table 4:** Literature reviews for large case series of CSF leakage of posterior fossa surgery.

Author	Year	N	Surgery	Dural Closing Method	Overall CSF Leakage Rate
Barker et al. [35]	1995	782	MVD surgery	Fascia, muscle	2.4%
Barker et al. [32]	1996	1336	MVD surgery	Fascia, muscle	1.5%
Samii et al. [7]	2002	143	MVD surgery	Muscle	4.8%
Steinbok et al. [36]	2007	174	Posterior fossa surgery	Synthetic dura, mibrin glue	33.3%
Park et al. * [6]	2007	678	MVD surgery	Muscle	0.3%
Than et al. [17]	2008	200	Posterior fossa surgery	Various methods	12.5%
Linskey et al. [37]	2008	36	MVD surgery	Fascia	2.8%
Narotam et al. [4]	2009	52	Posterior fossa surgery	Collagen matrix	3.8%
Bayazit et al. [38]	2009	412	Posterior fossa surgery	Primary closure	7.7%
Litvack et al. [14]	2009	150	Posterior fossa surgery	Collagen matrix	11.3%
Dubey et al. [39]	2009	500	Posterior fossa surgery	N/A	13.0%
Stoker et al. [8]	2012	100	MVD surgery	Synthetic dura, pericranium	17.0%
Tanrikulu et al. [16]	2016	50	MVD surgery	Collagen matrix	2%
Atlaf et al. [11]	2016	147	Posterior fossa surgery	N/A	17.0%
Kshettry et al. [2]	2016	84	Posterior fossa surgery	Collagen matrix	11.9%
Park et al. * [33]	2016	2040	MVD surgery	N/A	0.5%
Zhao et al. [40]	2017	1548	MVD surgery	N/A	1.6%
Venable et al. [34]	2018	86	Posterior fossa surgery	Primary suture	0%
Khan et al. [26]	2020	134	MVD surgery	Synthetic dura, histoacryl	3.7%
Lee at al. [41]	2020	122	Posterior fossa surgery	Primary closure, fibrin glue	27.0%
Inoue et al. [1]	2021	120	MVD surgery	Collagen matrix, fascia	4.2%
Present study	2023	475	MVD surgery	Synthetic dura, collagen matrix, fibrin glue	1.1%

* Our previous studies. N, number of patients; MVD, microvascular decompression; N/A, not available or not mentioned.

## Data Availability

All data included in this study can be provided by contacting hs5937@hanmail.net.
